# Potential Mechanisms of Biejiajian Pill in the Treatment of Diabetic Atherosclerosis Based on Network Pharmacology, Molecular Docking, and Molecular Dynamics Simulation

**DOI:** 10.1155/2022/3296279

**Published:** 2022-08-12

**Authors:** Rong Zhu, Bingzhao Du, Jiayao Yuan, Shuxun Yan, Mingyi Shao, Feng Sang, Qian Bi, Zhongrui Wang, Qian Zhen, Yu Fu

**Affiliations:** ^1^School of the First Clinical, Henan University of Chinese Medicine, Zhengzhou 450000, China; ^2^Department of Science Research, The First Affiliated Hospital of Henan University of Chinese Medicine, Zhengzhou 450000, China; ^3^Department of Endocrinology, The First Affiliated Hospital of Henan University of Chinese Medicine, Zhengzhou 450000, China; ^4^Department of Gastroenterology, The First Affiliated Hospital of Henan University of Chinese Medicine, Zhengzhou 450000, China; ^5^Department of Key Laboratory of Viral Diseases Prevention and Treatment of Traditional Chinese Medicine of Henan Province, The First Affiliated Hospital of Henan University of Chinese Medicine, Zhengzhou 450000, China

## Abstract

**Background:**

Biejiajian pill (BJJP), a classical traditional Chinese formula, has been reported that it has an effective treatment for diabetic atherosclerosis in recent years, but its underlying mechanisms remain elusive. The study aimed to explore the potential mechanisms of BJJP on diabetic atherosclerosis by integrating network pharmacology, molecular docking, and molecular dynamics simulation.

**Methods:**

The active components of BJJP were collected by TCMSP and TCMID, and then the potential targets were obtained from the SwissTargetPrediction database. The targets related to diabetic atherosclerosis were identified from the GeneCards and OMIM databases. The intersection of the potential targets regulated by active components of BJJP and the targets of diabetic atherosclerosis were common targets, which were visualized by the Venn diagram. The common targets were imported into the STRING database to construct a protein-protein interaction (PPI) network. The network of “Medicine-Compound-Target” was constructed with Cytoscape 3.7.1 software. GO functional enrichment analysis and KEGG pathway enrichment analysis were performed using the DAVID database and visualized through bioinformatics. The intersecting targets were input into Cytoscape 3.7.1 software, and the Network Analyzer tool was employed to screen out the key targets. Then molecular docking was used to verify the binding affinity between the active compounds and the key targets, and molecular dynamics simulation was used to investigate the stability of the binding models.

**Results:**

A total of 81 active components, 186 targets of BJJP, and 4041 targets of diabetic atherosclerosis were obtained. Furthermore, 121 overlapping targets were identified. GO functional enrichment analysis revealed that these targets were correlated with the oxidation-reduction process, negative regulation of apoptotic process, inflammatory response, and other biological processes. The results of the KEGG pathway enrichment analysis showed that the common targets mainly participated in proteoglycans in cancer, PPAR signaling pathway, adherens junction, insulin resistance, HIF-1 signaling pathway, PI3K-Akt signaling pathway, etc. The results of molecular docking confirmed that the core active components in BJJP could bind well to the key targets. Results from molecular dynamics simulation showed that the binding energies of AKT1-Luteolin, MMP9-quercetin, and MMP9-luteolin complexes were −28.93 kJ·mol^−1^, −37.12 kJ·mol^−1^, and −62.91 kJ·mol^−1^, respectively.

**Conclusion:**

The study revealed that BJJP is characterized as multicomponent, multitarget, and multipathway to treat diabetic atherosclerosis, which is helpful to provide ideas and a basis for pharmacological research and clinical application in the future.

## 1. Introduction

Diabetes mellitus has emerged as a pandemic. The International Diabetes Federation (IDF) has released new figures showing that 537 million adults are now living with diabetes worldwide, which is a rise of 16% (74 million) since the previous IDF estimates in 2019 [1]. The increased incidence rate is projected to continue as greater numbers of people adopt an unhealthy lifestyle [2]. Atherosclerosis is an important complication of diabetes and the leading cause of mortality in patients with diabetes [3]. Compared with nondiabetics, atherosclerosis in diabetic patients has the characteristics of high incidence, young age of onset, rapid disease progression, and multiple organs involved simultaneously [4]. This has emphasized the importance and urgency of studying the mechanism of diabetic atherosclerosis and exploring therapeutic options.

Biejiajian pill (BJJP) originated from *Synopsis of Golden Chamber*, a traditional Chinese medicine clinical prescription, is broadly utilized for the prevention and therapy of hepatocellular carcinoma [5], so the research on BJJP mainly focused on digestive disease. In recent years, some studies have found that BJJP has a significant effect on diabetic atherosclerosis. A randomized control method was used to find that BJJP can raise blood flow velocity in the lower limb, reduce low serum density lipoprotein cholesterol (LDL-C), total cholesterol (TC), and triglyceride (TG) levels, and improve the clinical symptoms of lower limb pain and numbness in lower extremity arteriosclerosis obliterans of diabetic patients [6]. Furthermore, previous studies have shown that BJJP has the effects of protecting vascular endothelial cells (VECs), inhibiting the proliferation and migration of vascular smooth muscle cells (VSMCs), and stabilizing plaques in diabetic atherosclerosis rats. The therapeutic potential may be achieved through regulating blood lipids, reducing inflammatory factors, lowering serum endothelin-1 (ET-1), increasing serum nitric oxide (NO) levels, reducing the expression of matrix metalloproteinases-9 (MMP-9) in aortic intima, and elevating APN-mRNA expression levels in adipose tissue [7–9]. However, its underlying mechanisms are not very clear, so it is necessary to explore the biological basis.

Diabetic atherosclerosis is a disease with a complex pathological mechanism and its potential mechanism remains elusive. At present, lifestyle interventions, antihyperglycemia, antihypertension, lipid regulation, and antiplatelet therapy are widely adopted in clinics, but single-targeted therapy cannot meet the needs of inhibiting the occurrence and development of diabetic atherosclerosis. Therefore, it is crucial that safer and more effective drugs are needed for the treatment of diabetic atherosclerosis.

Network pharmacology provides new horizons for traditional Chinese medicine (TCM) formula research. Its comprehensiveness, systematicness, and holistic concept are consistent with the TCM formula's characteristics of multicompound, multitarget, and multipathway. Most TCM formula prescriptions were derived from empiricism. It lacks scientific and rational mechanism interpretation although its efficacy has been validated for many years in clinical practice. The application of network pharmacology in the TCM formula is expected to achieve the change from experience-based medicine to evidence-based medicine, and network pharmacology is expected to become an important direction of TCM research studies [10].

**Figure 1:**
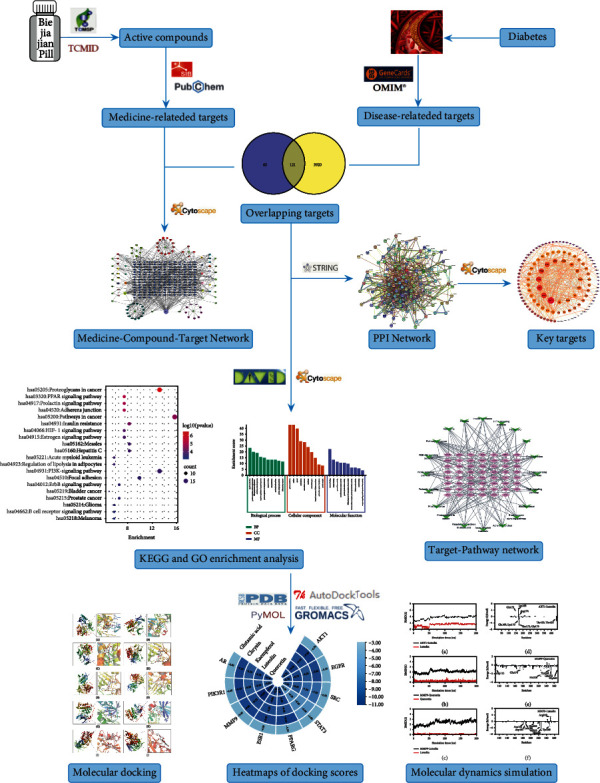
The flowchart of this study based on network pharmacology, molecular docking, and molecular dynamics simulation for deciphering the potential mechanisms of BJJP against diabetic atherosclerosis.

In this study, network pharmacology analysis, molecular docking techniques, and molecular dynamics simulation were used to visualize and elucidate the complex relationships and underlying mechanisms of BJJP in the treatment of diabetic atherosclerosis, which is helpful to provide a reference for experimental studies and clinical application in the future. The detailed flowchart of this study is summarized in Figure 1.

## 2. Materials and Methods

### 2.1. Screening of Active Compounds and Prediction of Corresponding Targets of BJJP

The active compounds of BJJP were acquired from the Traditional Chinese Medicine Systems Pharmacology database and Analysis Platform (TCMSP, http://tcmsp.com/tcmsp.php) [11] with the standards of oral bioavailability (OB) ≥ 30% and drug-likeness (DL) ≥ 0.18 and the Traditional Chinese Medicines Integrated Database (TCMID, http://47.100.169.139/tcmid/) [12] with the standards of the following two conditions simultaneously: firstly, GI absorption (gastrointestinal absorption) was “High”; secondly, two of the top five items of drug-likeness (including Lipinski, Ghose, Veber, Egan, and Muegge) were “Yes.” In addition, according to the published interrelated literature on compounds, the active compounds of unpredictable medicines were supplemented. Subsequently, the normalized name, PubChem CID, and the SDF structure format of the 2D structure of every active compound were obtained in the PubChem database (https://pubchem.ncbi.nlm.nih.gov) [13]. The targets were predicted by the SwissTargetPrediction database (http://www.swisstargetprediction.ch/) [14] and the related targets belonging to “Home sapiens” and “probability > 0.3” were chosen.

### 2.2. Construction of Medicine-Compound-Target Network

In order to more intuitively show the interactions between the active compounds of BJJP and their potential targets, the “medicine-compound-target” network was constructed by Cytoscape 3.7.1 software [15].

### 2.3. Screening of the Targets of Diabetic Atherosclerosis and Getting the Common Targets

The keyword “diabetic atherosclerosis” was used to extract the targets from the GeneCards (https://www.genecards.org/) [16] and Online Mendelian Inheritance in Man (OMIM, https://omim.org/) [17] databases. The targets of diabetic atherosclerosis belonging to “Homo sapiens” were integrated after deleting the repeated genes. A Venn diagram was drawn to get the overlapping targets between the drug targets and the disease targets.

### 2.4. Construction of the Protein-Protein Interaction (PPI) Network and Screening of the Key Targets

In general, it is difficult for a single protein to complete a series of complex biological processes. The common targets of BJJP and diabetic atherosclerosis were imported to the Search Tool for the Retrieval of Interacting Genes (STRING) database (https://string-db.org) [18] with the species limited to “Homo sapiens.” The information of target interaction was imported into Cytoscape 3.7.1 software, and the Network Analyzer tool was employed for topology analysis to screen out the key targets. The higher the degree, the larger the node and the coarser the edge, indicating a stronger relationship.

### 2.5. Gene Ontology (GO) Functional Enrichment and Kyoto Encyclopedia of Genes and Genomes (KEGG) Pathway Enrichment Analysis

The common targets were imported into the Database for Annotation Visualization and Integrated Discovery (DAVID, https://david.ncifcrf.gov/) [19] for bioinformation enrichment analysis, including biological process (BP), cellular component (CC), molecular function (MF), and KEGG pathway enrichment analysis. The GO terms and KEGG pathway terms with *P* value <0.05 were considered significant entries. A histogram was constructed with the first 10 functional categories of BP, CC, and MF. A bubble diagram was drawn by the top 20 signal pathways, and then the information of the first 20 pathways was imported into Cytoscape 3.7.1 software to display the “target-pathway” network.

### 2.6. Molecular Docking

The 3D structures of the key targets were retrieved and downloaded from the RCSB Protein Data Bank (https://www.rcsb.org/) [20], removing the ligands and water in PyMOL software [21], and the PBTQT formats were converted by AutoDock Tools1.5.6 software [22]. After that, utilize the AutoDock Vina to perform the molecular docking and get the docking scores. Visualization of the docking results was carried out on PyMOL software. Affinity could evaluate the binding capacity between the core active compounds and the targets. The affinity <−4.25 kcal/mol indicated that the compound could bind and interact with the target, whereas the affinity <−5.0 kcal/mol demonstrated good binding activity and the affinity <−7.0 kcal/mol demonstrated a strong binding force [23].

### 2.7. Molecular Dynamics Simulation

The docking models of the complexes on the top three molecular docking results were used as the initial conformation for molecular dynamics (MD) simulations. The force fields of the receptor were set to Amberff99SB using *pdb2gmx* in GROMACS v4.6.5 [24, 25]. Similarly, the force fields of the ligand were set to GAFF (general amber force field) using AmberTools 18 software [26, 27]. The complex systems were preprocessed by the following steps: firstly, the TIP3P water molecules solving the system with the dodecahedron box; secondly, Na^+^ and Cl^−^ neutralizing the system; and thirdly, the periodic boundary condition with a minimal distance of 1.2 nm. The steepest descent minimization method was applied to the energy minimization of complex systems. Meanwhile, 100 psNVT and NPT simulations were implemented to sequentially balance the complex systems. Furthermore, the PME (Particle Mesh Ewald), LINCS (Linear Constraint Solver), and SETTLE algorithms were performed to calculate the long-range electrostatic interactions, constrain hydrogen bonds, and restrict water molecules, respectively.

## 3. Results

### 3.1. Screening of the Active Compounds and Related Targets

A total of 81 active compounds in BJJP were screened via TCMSP and TCMID databases and the published related literature. The basic information of active ingredients is listed in Table S1 (Additional file 1). Then 186 related targets were predicted with the condition of “Homo sapiens” and “probability > 0.3” in the SwissTargetPrediction database.

### 3.2. The Network of Medicine-Compound-Target

**Figure 2:**
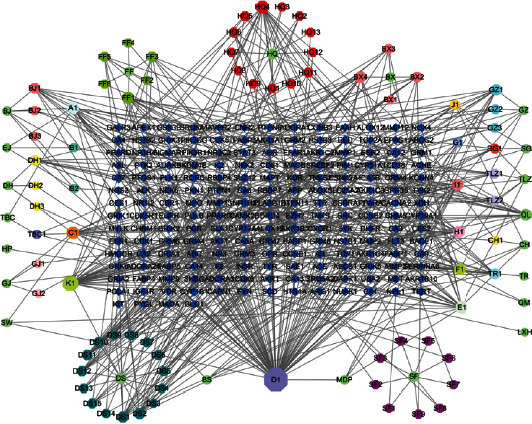
Medicine-compound-target network of BJJP. The area of the nodes represents their degree value. The bigger the area, the more important the node. BJ: Biejia; EJ: Ejiao; DH: Dahuang; TBC: Tubiechong; HP: Houpo; GJ: Ganjiang; SW: Shiwei; DS: Danshen; BS: Baishao; MDP: Mudanpi; SF: Shufu; LXH: Lingxiaohua; QM: Qumai; TR: Taoren; CH: Chaihu; QL: Qianglang; TLZ: Tinglizi; SG: Shegan; GZ: Guizhi; BX: Banxia; HQ: Huangqin; FF: Fengfang; XS: Xiaoshi. “A1” represents the common active ingredient of Biejia, Ejiao, and Tubiechong; “B1” and “B2” represent the common active ingredient of Biejia and Ejiao; “C1” represents the common active ingredient of Shiwei, Lingxiaohua, Banxia, Baishao, Dahuang, Ganjiang, Guizhi, Taoren, and Tinglizi; “D1” represents the common active ingredient of Shiwei, Chaihu, Mudanpi, and Tinglizi; “E1” represents the common active ingredient of Shiwei, Baishao, Chaihu, Mudanpi, and Tinglizi; “F1” represents the common active ingredient of Banxia, Chaihu, and Shegan; “G1” represents the common active ingredient of Baishao and Mudanpi; “H1” represents the common active ingredient of Baishao, Ganjiang, Mudanpi, and Guizhi; “I1” represents the common active ingredient of Baishao, Mudanpi, and Guizhi; “J1” represents the common active ingredient of Chaihu, Shegan, and Tinglizi; “K1” represents the common active ingredient of Shegan and Danshen.

To further reflect the interaction relationship between the active compounds and their targets, the network of medicine-compound-target was constructed through Cytoscape 3.7.1 software. The blue diamond nodes represented the targets, the green circle nodes represented Chinese medicine, and the octagon nodes represented the active compounds as shown in Figure 2.

### 3.3. Screening of the Targets Related to Diabetic Atherosclerosis and Getting the Common Targets

**Figure 3:**
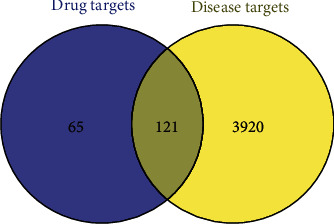
The Venn diagram showed the 121 overlapping targets between drug-related targets and disease-related targets.

For disease target identification, 3865 and 227 targets of “diabetic atherosclerosis” were identified from the GeneCards and OMIM databases, respectively. Subsequently, 4041 disease targets were integrated after deleting duplicates. Finally, 121 overlapping targets were shown as a Venn diagram between the drug targets and the disease targets (Figure 3).

### 3.4. Construction of PPI Network and Determination of the Key Targets

**Figure 4:**
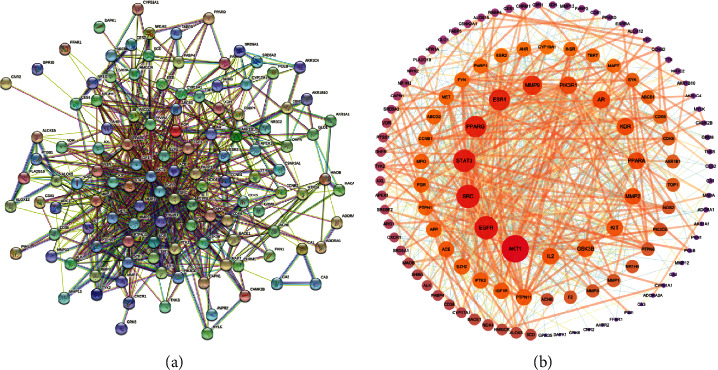
(a) The PPI network of the 121 common targets. (b) The selection of key targets via topology analysis.

The PPI network was constructed with a medium confidence score of 0.4 by importing the common targets into the STRING database, as shown in Figure 4(a). The network consisted of 121 nodes and 797 edges with an average node degree value of 13.2. Then the results after analysis were imported into Cytoscape 3.7.1 software. The degree values of common targets were computed through topological analysis and represented the interaction relationship between targets. The nodes of size and color were set according to their degree value and the size of nodes changing from big to small and the color from red to purple represented the degree from high to low, as shown in Figure 4(b). Ultimately, the top nine degree targets (AKT1, EGFR, SRC, STAT3, PPARG, ESR1, MMP9, PIK3R1, and AR) were identified as the key target proteins for further analysis.

### 3.5. GO and KEGG Enrichment Analysis

**Figure 5:**
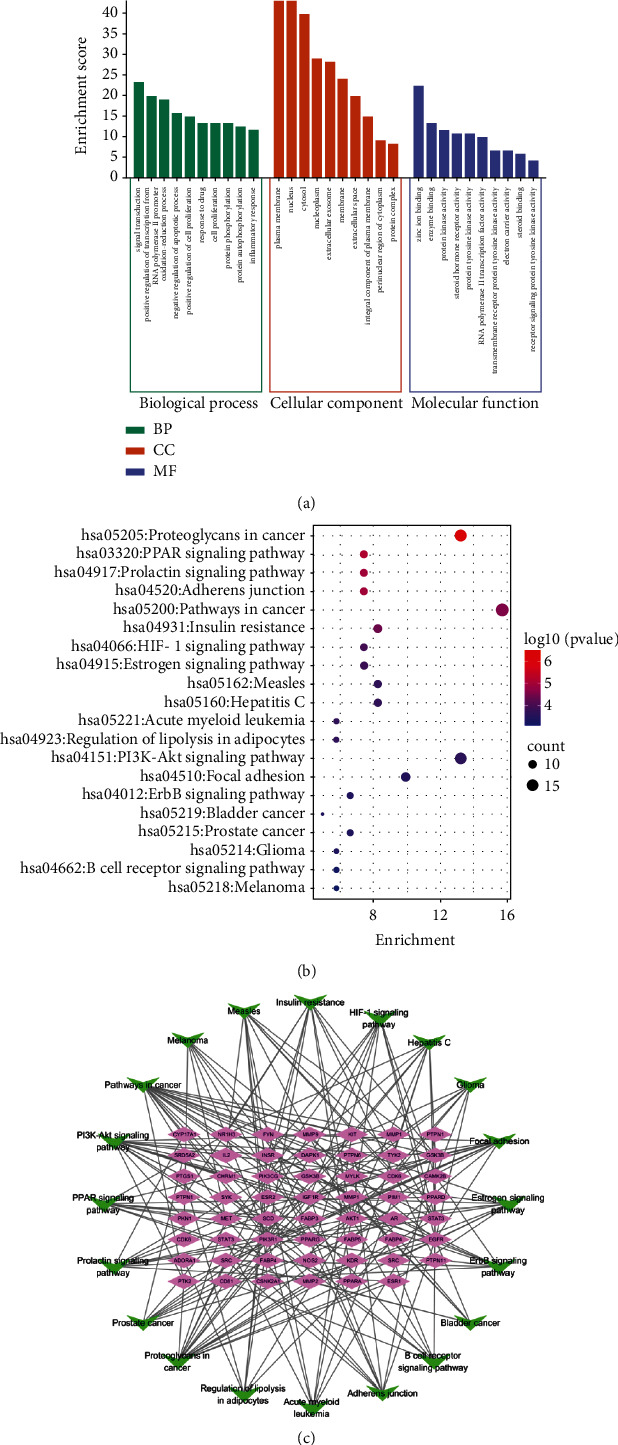
(a) Results of GO enrichment analysis. (b) Results of KEGG pathway analysis. (c) Target-pathway network of BJJP in the treatment of diabetic atherosclerosis.

GO function enrichment included BP, CC, and MF. Among them, 205 items were obtained via BP enrichment analysis, 31 items via CC, and 84 items via MF. The BP analysis indicated that the overlapping targets were mainly related to signal transduction, positive regulation of transcription from RNA polymerase II, oxidation-reduction process, negative regulation of the apoptotic process, inflammatory response, etc. The CC results mainly included plasma membrane, cytosol, extracellular space, protein complex, etc. The MF results are mainly comprised of enzyme binding, protein kinase activity, steroid binding, etc. The top 10 items of BP, CC, and MF were selected to draw the GO function histogram of BJJP in the treatment of diabetic atherosclerosis (Figure 5(a)). Meanwhile, the results of the KEGG analysis showed 63 pathways with a threshold value of *P* < 0.05 and a bubble diagram was drawn by the top 20 signal pathways. The larger the bubble, the more genes were gathered in the pathway. As depicted in Figure 5(b), the KEGG pathways of BJJP against diabetic atherosclerosis were mainly related to the PPAR signaling pathway, prolactin signaling pathway, adherens junction, insulin resistance, HIF-1 signaling pathway, and so on. In order to illuminate the correlation between the top 20 signal pathways and their targets, the network of targets-pathways was constructed, the purple nodes represented the targets, and the green represented the pathways (Figure 5(c)).

### 3.6. The Results of Molecular Docking

**Figure 6:**
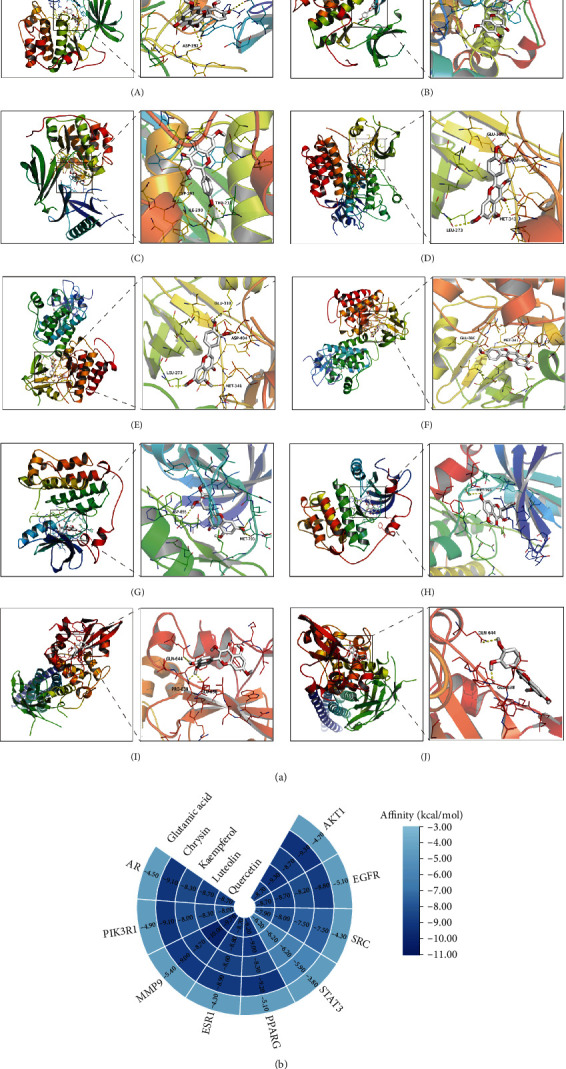
(a) Action modes of active compounds with key targets. (A) AKT1 and quercetin; (B) AKTI and luteolin; (C) AKT1 and kaempferol; (D) SRC and quercetin; (E) SRC and luteolin; (F) SRC and kaempferol; (G) EGFR and luteolin; (H) EGFR and kaempferol; (I) STAT3 and quercetin; (J) STAT3 and luteolin. (b) Heat maps of the docking scores for active compounds and key targets.

Molecular docking and binding ability prediction were carried out with five core active compounds (including quercetin, luteolin, kaempferol, chrysin, and glutamic acid) and nine key targets (AKT1, EGFR, SRC, STAT3, PPARG, ESR1, MMP9, PIK3R1, and AR), respectively. Finally, 45 groups of ligand-receptor docking results were obtained, 44 with affinity <−4.25 kcal/mol. The results of the molecular docking indicated most of the core active compounds could bind well with key targets and there were stable hydrogen bonds at the binding sites. A section of the docking patterns of the core compounds and targets are shown in Figure 6(a). The molecular docking scores is shown in Figure 6(b). The color from light to dark represented the binding ability from weak to strong. The results of molecular docking could provide data support for further experimental design of related traditional Chinese formulas or compounds. A portion of the binding affinity and the number of hydrogen bonds were shown in Table 1.

### 3.7. The Results of MD Simulation

**Figure 7:**
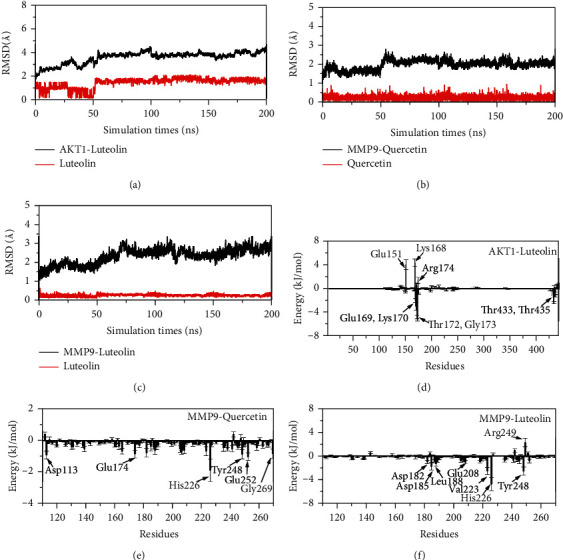
(a–c) The RMSD of AKT1-luteolin, MMP9-quercetin, and MMP9-luteolin. (d–f) The residues energy decomposition of AKT1-luteolin, MMP9-quercetin, and MMP9-luteolin complex systems.

The MD simulations of the AKT1-luteolin, MMP9-quercetin, and MMP9-luteolin complexes were implemented to investigate the stability of the binding models. From Figure 7(a), it was observed that the AKT1-luteolin complex has already reached equilibrium at about 50 ns with 4.0 Å RMSD. From Figures 7(b) and 7(c), it was seen that MMP9-quercetin and MMP9-luteolin complexes have already reached equilibrium at about 50 ns and 70 ns with 2.5 Å and 3.0 Å RMSD, respectively. These results suggested that luteolin could bind efficiently to AKT1 and MMP9 and quercetin could bind well to MMP9.

Meanwhile, the binding energies of these complexes were calculated using the MM/PBSA method. From Table 2, the binding energies of AKT1-luteolin, MMP9-quercetin, and MMP9-luteolin complexes were −28.93 kJ·mol^−1^, −37.12 kJ·mol^−1^, and −62.91 kJ·mol^−1^, respectively, showing that the binding processes were spontaneous. In addition, it also implied that luteolin was more likely to bind to MMP9 compared to quercetin, which was consistent with the docking result.

Then, the binding energies of these complexes were decomposed to each residue. From Figure 7(d), it was observed that residues Glu169, Lys170, Thr172, Gly173, Thr433, and Thr435 were helpful for binding between AKT1 and luteolin, while residues Glu151, Lys168, and Arg174 were the opposite. From Figure 7(e), it was found that residues Asp113, Glu174, His226, Tyr248, Glu252, and Gly269 played a significant role in the binding process between quercetin and MMP9. From Figure 7(f), it was seen that residues Asp182, Asp185, Leu188, Glu208, Val223, His226, Tyr248, and Arg249 were beneficial to the binding process between luteolin and MMP9.

## 4. Discussion

Diabetes mellitus (DM) is a chronic disease that occurs when the pancreas is no longer able to make insulin or when the body cannot make good use of the insulin it produces. People with diabetes have an increased risk of developing a number of serious health problems. Notably, macrovascular complications are the leading cause of disability and death in patients with diabetes mellitus, and the injury of VECs is the initiating factor and the key link. Previous studies have shown that BJJP has a significant effect on diabetic atherosclerosis. However, the underlying mechanisms of BJJP against diabetic atherosclerosis are not fully elucidated. In this study, we employ the technology of network pharmacology, approaches of molecular docking, and MD simulation to explore the potential mechanisms of BJJP on diabetic atherosclerosis in order to better guide clinical treatment.

Firstly, we retrieved the active compounds of BJJP, and 81 were identified. Then we constructed the medicine-compound-target network by mapping 81 active compounds to 186 related targets. Notably, the top five active compounds with high degree value are quercetin, luteolin, kaempferol, chrysin, and glutamic acid, respectively, indicating that they may be the core bioactive compounds. Among them, quercetin, luteolin, kaempferol, and chrysin all belong to flavonoids, which exist widely in nature, and have the function of both medicine and food and a wide range of pharmacological activities. They all show antioxidative and anti-inflammatory effects; however, the mechanisms are not exactly the same. To some extent, these were associated with the pathogenesis of diabetic atherosclerosis. It has been reported that quercetin may have promising potential in ameliorating diabetic atherosclerotic pathophysiology in the diabetic-induced atherosclerotic rat model carotid artery induced by the administration of a high-fat diet with streptozotocin by inhibiting oxidative stress and inflammatory responses mechanistically by modulating the AMPK/SIRT1/NF-*κ*B signaling pathway [28]. Luteolin and chrysin can combine with the hydrogen atom of the phenolic hydroxyl group to form the free radical of flavonoid, which can play the role of antioxidation. At the same time, it showed a better anti-inflammatory effect [29]. VSMC proliferation and migration, as well as endothelial cell apoptosis, are important processes that participate in the pathogenesis of diabetic atherosclerosis. Luteolin can inhibit proliferation and migration in VSMCs through downregulation of the PI3K/Akt signaling pathway in endothelial cells, regulating the MAPK signaling pathway and inhibiting the generation of reactive oxygen species (ROS) in order to inhibit the occurrence and development of diabetic atherosclerosis [30]. Kaempferol has been demonstrated to provide benefits for the treatment of atherosclerosis, coronary heart disease, hyperlipidemia, and diabetes through its antioxidant and anti-inflammatory properties by the inhibition of the ASK1/MAPK signaling pathway and the regulation of oxidative stress [31].

Metformin is currently recommended as a first-line treatment for patients with diabetes and as an essential drug in combination, in the guides of both domestic and international. Its main pharmacological effect is to reduce blood sugar by reducing the liver glucose output and improving insulin resistance. Metformin alone did not increase the risk of hypoglycemia and reduced the risk of cardiovascular events, but there were gastrointestinal adverse events [32]. Chrysin is one of the main components of Huangqin, and it could produce similar effects as metformin verified in nude diabetic mice models. It could decrease glucose and triglyceride levels and improve the generation of proinflammatory cytokines involved in the development of diabetes and its complications, such as diabetic atherosclerosis and other cardiovascular diseases [33]. In epidemiologic studies, abnormal metabolism of glutamic acids has been shown to be related to insulin resistance, type 2 diabetes, and cardiovascular disorders. Genetic locus associated among type 2 diabetic patients may affect the risk of coronary heart disease by affecting the metabolism of glutamic acids in endothelial cells.

Insulin resistance is closely associated with chronic low-grade inflammation through interactions with the insulin signaling pathway in the liver and adipose tissue, which is also considered one of the main factors responsible for the onset and progression of diseases such as obesity, diabetes, and atherosclerosis. Therefore, the improvement of insulin resistance is important in the management of diabetes atherosclerosis. The HIF-1 signaling pathway plays a key role in the development of atherosclerosis through cell-specific responses acting on endothelial cells, VSMCs, and macrophages. Through the upregulation of VEGF, NO, ROS, and PDGF, it is able to cause endothelial cell dysfunction, proliferation, angiogenesis, and inflammation [34]. It was also found that the TNF, PI3K/Akt, and MAPK signal pathways can interact with the HIF signal pathway, leading to the gradual progress of diabetes and its complications [35]. Therefore, the HIF-1 signaling pathway may be an effective way to treat diabetic atherosclerosis to a certain extent. The PPAR signaling pathway regulates glucose and lipid metabolism, endothelial function, and inflammation [36]. Peroxisome proliferator-activated receptors (PPARs) are a subgroup of the nuclear hormone receptor superfamily of ligand-activated transcription factors, which play an important role in the pathogenesis of type 2 diabetes mellitus (T2DM) and atherosclerosis. It has been reported that genetic disruption or pharmacological inhibition of the AT1R attenuates atherosclerosis and improves endothelial function in diabetic ApoE^−/−^ mice via the PPAR*γ* pathway [37]. The functions of the prolactin receptor have been extended to include islet differentiation, adipocyte control, and immune modulation. Also, the prolactin signaling pathway disruption has been involved in diabetes. Meanwhile, prolactin has been suggested to stimulate the development of atherosclerosis and cardiovascular disease through its effects on metabolism and inflammation, and the inhibition of prolactin function may beneficially affect atherosclerosis burden [38]. Adherens junction is one of the aspects of the integrity of vascular endothelial function. A study showed that altering adherens junction phosphorylation levels can impair vascular endothelial permeability and affect the binding and dissociation of its components in high-glucose conditions, which will be helpful for understanding the process of arterial injury in diabetes patients and might open up new targets for therapeutic development [39].

In the PPI network, we found that AKT1 (degree = 70), EGFR (degree = 53), SRC (degree = 53), STAT3 (degree = 50), PPARG (degree = 45), ESR1 (degree = 44), MMP9 (degree = 37), PIK3R1 (degree = 29), and AR (degree = 29) played important roles in BJJP against diabetic atherosclerosis. It is well known that AKT1 is one of the three isoforms of the AKT family. AKT is one of the key molecules activated downstream of the PI3kinase signaling pathway and regulates many cellular processes including metabolism, proliferation, cell survival, growth, and angiogenesis through serine and/or threonine phosphorylation of a range of downstream substrates. However, one of the key roles of AKT is the regulation of glucose uptake into insulin-responsive tissues, which is mediated by the translocation of insulin-regulated glucose transporter 4 (GLUT4) from vesicular intracellular compartments to the plasma membrane [40]. In addition, AKT also plays a role in the repression of liver gluconeogenesis by insulin by suppressing the expression of phosphoenolpyruvate carboxykinase and glucose 6-phosphatase in this process [41]. Nitric oxide (NO) is mainly produced by endothelial cells and acts as a vasodilator and inhibitor of smooth muscle cell migration and platelet activation, thereby protecting from vascular disease. Insulin is known to affect the vasculature by increasing the release of NO from endothelial cells through the PI3K/AKT pathway. The cells where AKT contributes to cardiovascular function are endothelial cells, which predominantly express AKT1 [42]. Genetically modified mice revealed the protective role of AKT1 in vascular function, with AKT1 KO mice on an ApoE^−/−^ background displaying severe peripheral vascular disease, atherosclerosis, occlusive coronary artery disease, plaque vulnerability, and cardiac dysfunction. This manifested itself in decreased blood flow, reduced migration of fibroblasts and endothelial cells, and decreased production of the potent vasodilator NO [43, 44]. EGFR, which is distributed in all vascular epithelial cells, can interfere with cell growth, differentiation, and proliferation by participating in RAS/RAF/MEK/MAPK and PI3K/PDK1/AKT pathways. It is reported that enhanced EGFR phosphorylation and its downstream ER stress are involved in cardiac fibrosis and vascular endothelial dysfunction in type I diabetes mellitus [45]. SRC is a tyrosine-protein kinase that regulates cellular metabolism, survival, and proliferation. Studies have shown that SRC kinase plays an important role in RAGE-mediated inflammatory gene expression, migration in VSMCs, and key events associated with diabetic vascular complications [46]. Activation of signal transducers and activators of transcription (STAT) pathway by hyperglycemia and dyslipidemia contributes to the progression of diabetic atherosclerosis and other diabetic complications. Recio had proved inhibition of STAT1/STAT3 activation and target gene expression in VSMCs and macrophages could suppress cytokine-induced cell migration and adhesion processes [47]. PPARG could not only inhibit the expression of inflammatory factors such as TNF-*α* and IL-1 but also regulate adipocyte differentiation as well as lipid metabolism. This activity might contribute to the treatment of diabetic atherosclerosis [48]. Previously, an African-American case-control study detected an association between a region of the ESR1 gene and type 2 diabetes. Moreover, it appeared that ESR1 contributes to type 2 diabetes and CVD risk via pleiotropic effects, leading to insulin resistance, a poor lipid profile, and obesity [49]. MMP9 is a member of the metalloproteinase family and one of the most important enzymes involved in matrix metabolism. Matrix metalloproteinases (MMPs), a large family of zinc and calcium-dependent endopeptidases that degrade all components of the extracellular matrix and basement membrane proteins, are known to be closely associated with diabetic atherosclerosis. Inhibition of the I*κ*B/NF-*κ*B signal pathway via the MMP9/TIMP1 axis has an effect on vascular inflammation T2DM atherosclerosis [50]. It can also promote the consumption and rupture of fibrous cap and regulate the stability of plaque. It has been found that PIK3R1 can affect insulin signaling transduction and thus play a direct role in the development of diabetes through insulin resistance signaling [51]. Accumulating data emphasize the importance of AR on the expression in the key vascular tissues, including endothelial cells and VSMCs [52]. It was also verified that AR was crucial in mediating the effects of androgens to regulate glucose and lipid metabolisms in males and relatively early coronary atherosclerosis [53].

In conclusion, the therapeutic potential of BJJP in the treatment of diabetic atherosclerosis may be through regulating blood lipids, reducing inflammatory factors and oxidation-reduction reaction, and so on.

Molecular docking and MD simulation were employed to further verify the results obtained. The results of the molecular docking indicated most of the core active compounds could bind well with key targets. Then the results of molecular dynamics simulation suggested that AKT1-luteolin, MMP9-quercetin, and MMP9-luteolin could bind tightly, and the binding energies of the complexes were −28.93 kJ·mol^−1^, −37.12 kJ·mol^−1^, and −62.91 kJ·mol^−1^, respectively, showing that the binding processes were spontaneous.

However, it should be noted that there were certain limitations of the network pharmacology. Firstly, it is difficult for current network pharmacology technology to achieve the goal of quantizing, such as the dose-efficacy relationship between the drugs and disease [10]. Secondly, its network modeling is based on the information of the existing databases and existing experiments data; therefore, a part of the compounds and targets of insects are not found and included. Thirdly, the study based on network pharmacology is a static network analysis while the occurrence and development of disease and the action of drugs are both dynamic processes, so the following experiments in vivo or in vitro can be carried out on this basis to explore the deeper mechanism of BJJP in the treatment of diabetic atherosclerosis. Despite the limitations of this study, the results revealed the underlying mechanisms of BJJP against diabetic atherosclerosis, which had certain clinical value and research significance.

## 5. Conclusion

In conclusion, the study was based on network pharmacology, molecular docking, and MD simulation to explore the potential mechanisms of BJJP against diabetic atherosclerosis through compound screening, target prediction, construction of PPI network, GO enrichment analysis, and KEGG pathway enrichment analysis. The results indicated that quercetin, luteolin, kaempferol, chrysin, glutamic acids, and other effective compounds of BJJP showed therapeutic effects against diabetic atherosclerosis via multiple targets and multiple pathways. In addition, the good activities of the core active compounds and the key targets were verified by the molecular docking method and molecular dynamics simulation. All in all, the results of this study provided a reference for clinical application and guidance for further experimental verification of BJJP for the treatment of diabetic atherosclerosis.

## Figures and Tables

**Table 1:** Docking affinity and relevant results of hub targets and key compounds.

Compound	Compound 2D structure	Target	PDB ID	Structure with initial ligand	Affinity (kcal/mol)	Amino acid residue	The number of hydrogen bonds
Quercetin	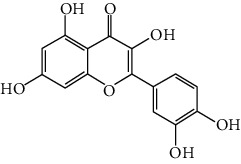	AKT1	6HHF	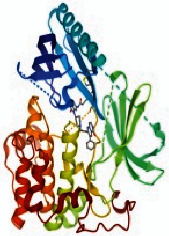	−8.7	ASP292, VAL271, GLN79	3
Luteolin	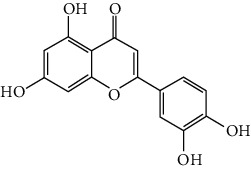	AKT1	6HHF	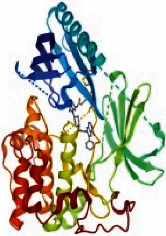	−9.3	THR211, THR291	2
Kaempferol	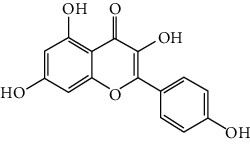	AKT1	6HHF	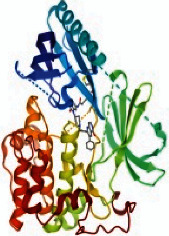	−8.7	ILE290, ASP292, THR211	3
Quercetin	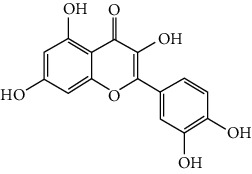	SRC	4MXY	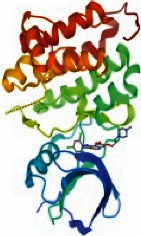	−7.9	GLU310, ASP404, MET341, LEU273	5
Luteolin	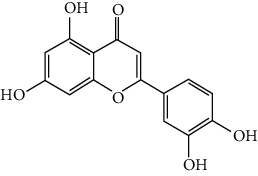	SRC	4MXY	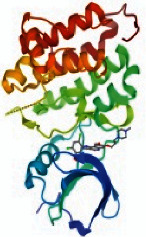	−8.0	ASP404, LEU273, GLU310, MET341	4
Kaempferol	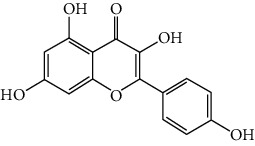	SRC	4MXY	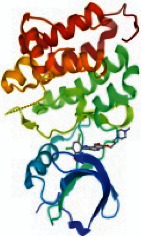	−7.5	MET341, GLU310	3
Luteolin	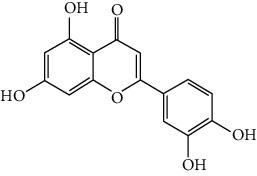	EGFR	3W2S	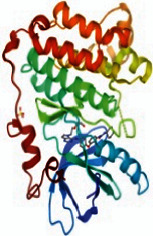	−8.7	ASP855, MET793	2
Kaempferol	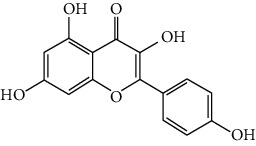	EGFR	3W2S	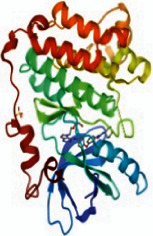	−8.2	MET793	1
Quercetin	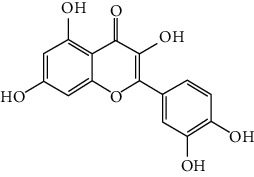	STAT3	6NJS	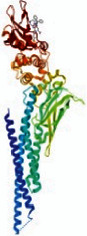	−6.2	GLU638, GLN644, PRO639	3
Luteolin	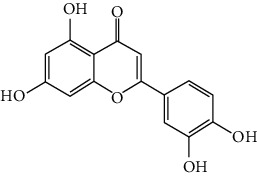	STAT3	6NJS	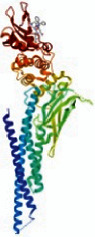	−6.2	GLU638, GLN644	2

**Table 2:** The binding free energy of AKT1-luteolin, MMP9-quercetin, and MMP9-luteolin complex systems by the MM/PBSA method.

Systems	Δ*E*_vdw_ (kJ·mol^−1^)	Δ*E*_ele_ (kJ·mol^−1^)	Δ*G*_polar_ (kJ·mol^−1^)	Δ*G*_nonpolar_ (kJ·mol^−1^)	Δ*E*_binding_ (kJ·mol^−1^)
AKT1-luteolin	−87.36 ± 2.03	−56.16 ± 3.13	123.85 ± 3.96	−9.27 ± 0.16	−28.93 ± 2.93
MMP9-quercetin	−22.95 ± 8.53	−7.58 ± 2.41	−4.67 ± 8.05	−1.92 ± 0.79	−37.12 ± 4.23
MMP9-luteolin	−85.32 ± 2.06	−20.79 ± 4.71	50.20 ± 1.12	−7.00 ± 1.57	−62.91 ± 8.23

*Note.* Δ*E*_vdw_, Δ*E*_ele_, Δ*G*_polar_, Δ*G*_nonpolar_, and Δ*E*_binding_ are van der Waals contribution, electrostatic contribution, polar solvation energy, nonpolar solvation energy, and binding energy, respectively.

## Data Availability

The data for this study can be provided by the corresponding author (Yu Fu: kybfuyu@126.com).

## Supplementary Materials

Supplementary Materials Additional file 1: Table S1: basic information of part of active compounds of BJJP.
