# Supplementary material for: Potential Mechanisms of Biejiajian Pill in the Treatment of Diabetic Atherosclerosis Based on Network Pharmacology, Molecular Docking, and Molecular Dynamics Simulation
Source: Evid Based Complement Alternat Med. 2022 Aug 12;2022:3296279. doi: 10.1155/2022/3296279 (PMC9391107; doi:10.1155/2022/3296279)
Supplement: Supplementary Materials — Additional file 1: Table S1: basic information of part of active compounds of BJJP. [file 3296279.f1.docx]

TABLE S1: Basic information of part of active compounds of BJJP

| Number | PubChem CID | Compound | Canonical Smiles |
| --- | --- | --- | --- |
| BJ3 | 12895043 | 24,25-Dihydroxyvitamin D | CC(CCC(C(C)(C)O)O)C1CCC2C1(CCCC2=CC=C3CC(CCC3=C)O)C |
| BJ1 | 5460221 | Oleate | CCCCCCCCC=CCCCCCCCC(=O)[O-] |
| BJ2 | 53477803 | Heptadecanoyl carnitine | CCCCCCCCCCCCCCCCC(=O)OC(CC(=O)[O-])C[N+](C)(C)C |
| B1 | 6140 | Phenylalanine | C1=CC=C(C=C1)CC(C(=O)O)N |
| A1 | 611 | DL-Glutamic acid | C(CC(=O)O)C(C(=O)O)N |
| B2 | 876 | DL-Methionine | CSCCC(C(=O)O)N |
| C1 | 222284 | Beta-Sitosterol | CCC(CCC(C)C1CCC2C1(CCC3C2CC=C4C3(CCC(C4)O)C)C)C(C)C |
| D1 | 5280343 | Quercetin | C1=CC(=C(C=C1C2=C(C(=O)C3=C(C=C(C=C3O2)O)O)O)O)O |
| E1 | 5280863 | Kaempferol | C1=CC(=CC=C1C2=C(C(=O)C3=C(C=C(C=C3O2)O)O)O)O |
| HQ1 | 70699355 | Stigmasteryl glucoside | CCC(C=CC(C)C1CCC2C1(CCC3C2CC=C4C3(CCC(C4)OC5C(C(C(C(O5)CO)O)O)O)C)C)C(C)C |
| HQ4 | 25200543 | 7-Hydroxy-4-oxo-2-phenylchromen-5-olate | C1=CC=C(C=C1)C2=CC(=O)C3=C(C=C(C=C3O2)O)[O-] |
| HQ3 | 25200626 | 6,8-Dihydroxy-9-oxoxanthen-4-olate | C1=CC2=C(C(=C1)[O-])OC3=CC(=CC(=C3C2=O)O)O |
| HQ6 | 5322065 | 7,3',4'-Trihydroxyflavone | C1=CC(=C(C=C1C2=CC(=O)C3=C(O2)C=C(C=C3)O)O)O |
| HQ2 | 25203524 | 4',5-Dihydroxy-3'-methoxyflavon-7-olate(1-) | COC1=C(C=CC(=C1)C2=CC(=O)C3=C(C=C(C=C3O2)O)O)[O-] |
| HQ13 | 31161 | Pedalitin | COC1=C(C(=C2C(=C1)OC(=CC2=O)C3=CC(=C(C=C3)O)O)O)O |
| HQ9 | 5318276 | 1-(4-Hydroxy-3-methoxyphenyl)octane-3,5-diol | CCCC(CC(CCC1=CC(=C(C=C1)O)OC)O)O |
| HQ7 | 5321859 | 5,7,3',4'-Tetrahydroxy-6,8-dimethoxyflavone | COC1=C(C(=C2C(=C1O)C(=O)C=C(O2)C3=CC(=C(C=C3)O)O)OC)O |
| HQ5 | 440735 | Eriodictyol | C1C(OC2=CC(=CC(=C2C1=O)O)O)C3=CC(=C(C=C3)O)O |
| HQ11 | 5281665 | Isoscutellarein | C1=CC(=CC=C1C2=CC(=O)C3=C(O2)C(=C(C=C3O)O)O)O |
| HQ8 | 5320315 | Oroxylin A | COC1=C(C2=C(C=C1O)OC(=CC2=O)C3=CC=CC=C3)O |
| HQ10 | 5281703 | Wogonin | COC1=C(C=C(C2=C1OC(=CC2=O)C3=CC=CC=C3)O)O |
| HQ12 | 188316 | 5-Hydroxy-7,8-dimethoxyflavone | COC1=C(C2=C(C(=C1)O)C(=O)C=C(O2)C3=CC=CC=C3)OC |
| FF6 | 69988 | 2-Methoxyhydroquinone | COC1=C(C=CC(=C1)O)O |
| FF5 | 338 | Salicylic acid | C1=CC=C(C(=C1)C(=O)O)O |
| FF4 | 114973 | Benzoylphenylalanyl-alanyl-proline | CC(C(=O)N1CCCC1C(=O)O)NC(=O)C(CC2=CC=CC=C2)NC(=O)C3=CC=CC=C3 |
| FF3 | 54675866 | 3,4-Dihydroxybenzoate | C1=CC(=C(C=C1C(=O)O)O)[O-] |
| FF2 | 10773058 | 2,6-Dideuterio-4-(2-hydroxyethyl)phenol | C1=CC(=CC=C1CCO)O |
| FF1 | 54710367 | 3-(3,4-Dihydroxyphenyl)prop-2-enoate | C1=CC(=C(C=C1C=CC(=O)O)O)[O-] |
| QM | 71719485 | (3R,4S,4Ar,6aR,6bS,8aS,12aS,14aR,14bR)-3-hydroxy-4,6a,6b,11,11,14b-hexamethyl-1,2,3,4a,5,6,7,8,9,10,12,12a,14,14a-tetradecahydropicene-4,8a-dicarboxylic acid | CC1(CCC2(CCC3(C(=CCC4C3(CCC5C4(CCC(C5(C)C(=O)O)O)C)C)C2C1)C)C(=O)O)C |
| SF1 | 5997 | Cholesterol | CC(C)CCCC(C)C1CCC2C1(CCC3C2CC=C4C3(CCC(C4)O)C)C |
| SF2 | 5998 | (9S,14S,17R)-13-Methyl-6,7,8,9,11,12,14,15,16,17-decahydrocyclopenta[a]phenanthrene-3,17-diol | CC12CCC3C(C1CCC2O)CCC4=C3C=CC(=C4)O |
| SF3 | 5999 | 2-[(1R,2R,3S,4R,5R,6S)-3-(Diaminomethylideneamino)-4-[(2S,3S,4S,5R)-3-[(2R,3R,4R,5S,6R)-4,5-dihydroxy-6-(hydroxymethyl)-3-(methylamino)oxan-2-yl]oxy-4-formyl-4-hydroxy-5-methyloxolan-2-yl]oxy-2,5,6-trihydroxycyclohexyl]guanidine | CC1C(C(C(O1)OC2C(C(C(C(C2O)O)N=C(N)N)O)N=C(N)N)OC3C(C(C(C(O3)CO)O)O)NC)(C=O)O |
| SF4 | 6000 | Tubocurarine chloride | CN1CCC2=CC(=C3C=C2C1CC4=CC=C(C=C4)OC5=C6C(CC7=CC(=C(C=C7)O)O3)[N+](CCC6=CC(=C5O)OC)(C)C)OC |
| SF5 | 6001 | 7,12-Dimethylbenzanthracene | CC1=C2C=CC3=CC=CC=C3C2=C(C4=CC=CC=C14)C |
| SF6 | 6002 | Ammonium,ethyldimethyl(2-phenothiazin-10-ylpropyl)-,ethyl sulfate | CC[N+](C)(C)CC(C)N1C2=CC=CC=C2SC3=CC=CC=C31.CCOS(=O)(=O)[O-] |
| SF7 | 6003 | Ethyl-dimethyl-(2-phenothiazin-10-ylpropyl)azanium | CC[N+](C)(C)CC(C)N1C2=CC=CC=C2SC3=CC=CC=C31 |
| SF8 | 6004 | Ethyl sulfate | CCOS(=O)(=O)O |
| SF9 | 6005 | Apomorphine | CN1CCC2=C3C1CC4=C(C3=CC=C2)C(=C(C=C4)O)O |
| BX4 | 5484202 | Stigmast-4-en-3-one | CCC(CCC(C)C1CCC2C1(CCC3C2CCC4=CC(=O)CCC34C)C)C(C)C |
| F1 | 5280794 | Stigmasterol | CCC(C=CC(C)C1CCC2C1(CCC3C2CC=C4C3(CCC(C4)O)C)C)C(C)C |
| BX3 | 5281605 | Baicalein | C1=CC=C(C=C1)C2=CC(=O)C3=C(O2)C=C(C(=C3O)O)O |
| BX2 | 5282768 | 11-Eicosenoic acid | CCCCCCCCC=CCCCCCCCCCC(=O)O |
| BX1 | 11438306 | Cyclo(L-tyrosyl-L-phenylalanyl) | C1=CC=C(C=C1)CC2C(=O)NC(C(=O)N2)CC3=CC=C(C=C3)O |
| DS1 | 5281330 | Poriferasterol | CCC(C=CC(C)C1CCC2C1(CCC3C2CC=C4C3(CCC(C4)O)C)C)C(C)C |
| DS2 | 457801 | Clionasterol | CCC(CCC(C)C1CCC2C1(CCC3C2CC=C4C3(CCC(C4)O)C)C)C(C)C |
| DS3 | 3083514 | Danshenol A | CC1COC2=C1C(=O)C(C3=C2C=CC4=C(C=CC=C43)C)(CC(=O)C)O |
| DS4 | 3083515 | Danshenol B | CC1COC2=C1C(=O)C(C3=C2C=CC4=C3CCCC4(C)C)(CC(=O)C)O |
| DS5 | 9841799 | (2R)-3-(3,4-Dihydroxyphenyl)-2-[(Z)-3-(3,4-dihydroxyphenyl)prop-2-enoyl]oxypropanoic acid | C1=CC(=C(C=C1CC(C(=O)O)OC(=O)C=CC2=CC(=C(C=C2)O)O)O)O |
| DS6 | 14609847 | 10,11-Dihydro-1-methyl-10,11-dioxophenanthro[1,2-b]furan-6-carbaldehyde | CC1=COC2=C1C(=O)C(=O)C3=C2C=CC4=C(C=CC=C43)C=O |
| DS7 | 160254 | Cryptotanshinone | CC1COC2=C1C(=O)C(=O)C3=C2C=CC4=C3CCCC4(C)C |
| DS8 | 164676 | Tanshinone IIA | CC1=COC2=C1C(=O)C(=O)C3=C2C=CC4=C3CCCC4(C)C |
| DS9 | 341 | Digallic acid | C1=C(C=C(C(=C1O)O)O)C(=O)OC2=CC(=CC(=C2O)O)C(=O)O |
| DS10 | 10470747 | (6S,7R)-6,7-Dihydroxy-1,6-dimethyl-8,9-dihydro-7H-naphtho[1,2-g][1]benzofuran-10,11-dione | CC1=COC2=C1C(=O)C(=O)C3=C2C=CC4=C3CCC(C4(C)O)O |
| DS11 | 5321620 | Tanshindiol B | CC1=COC2=C1C(=O)C(=O)C3=C2C=CC4=C3CCC(C4(C)O)O |
| DS12 | 126072 | Tanshindiol C | CC1=COC2=C1C(=O)C(=O)C3=C2C=CC4=C3CCC(C4(C)O)O |
| DS13 | 9926694 | (S)-6-(Hydroxymethyl)-1,6-dimethyl-6,7,8,9-tetrahydrophenanthro[1,2-b]furan-10,11-dione | CC1=COC2=C1C(=O)C(=O)C3=C2C=CC4=C3CCCC4(C)CO |
| K1 | 5280445 | Luteolin | C1=CC(=C(C=C1C2=CC(=O)C3=C(C=C(C=C3O2)O)O)O)O |
| DS15 | 40785034 | 3,9-Dimethyl-2,3-dihydrophenanthro[1,2-b]furan-4,5-dione | CC1COC2=C1C(=O)C(=O)C3=C2C=CC4=C(C=CC=C43)C |
| DS14 | 160142 | Miltirone | CC(C)C1=CC2=C(C3=C(C=C2)C(CCC3)(C)C)C(=O)C1=O |
| G1 | 64971 | Betulinic acid | CC(=C)C1CCC2(C1C3CCC4C5(CCC(C(C5CCC4(C3(CC2)C)C)(C)C)O)C)C(=O)O |
| H1 | 12303645 | 3-Epi-beta-Sitosterol | CCC(CCC(C)C1CCC2C1(CCC3C2CC=C4C3(CCC(C4)O)C)C)C(C)C |
| I1 | 9064 | Cianidanol | C1C(C(OC2=CC(=CC(=C21)O)O)C3=CC(=C(C=C3)O)O)O |
| DH2 | 11968447 | 1,6-Dihydroxy-3-methyl-8-[(2R,3S,4R,5R,6S)-3,4,5-trihydroxy-6-(hydroxymethyl)oxan-2-yl]oxyanthracene-9,10-dione | CC1=CC2=C(C(=C1)O)C(=O)C3=C(C2=O)C=C(C=C3OC4C(C(C(C(O4)CO)O)O)O)O |
| DH3 | 442762 | Physcion 8-gentiobioside | CC1=CC2=C(C(=C1)O)C(=O)C3=C(C2=O)C=C(C=C3OC4C(C(C(C(O4)COC5C(C(C(C(O5)CO)O)O)O)O)O)O)OC |
| DH1 | 73160 | (-)-Catechin | C1C(C(OC2=CC(=CC(=C21)O)O)C3=CC(=C(C=C3)O)O)O |
| TBC1 | 5281617 | Genkwanin | COC1=CC(=C2C(=C1)OC(=CC2=O)C3=CC=C(C=C3)O)O |
| CH1 | 389001 | 3',4',5',3,5,6,7-Heptamethoxyflavone | COC1=CC(=CC(=C1OC)OC)C2=C(C(=O)C3=C(C(=C(C=C3O2)OC)OC)OC)OC |
| J1 | 5281654 | Isorhamnetin | COC1=C(C=CC(=C1)C2=C(C(=O)C3=C(C=C(C=C3O2)O)O)O)O |
| GJ1 | 5281698 | Sexangularetin | COC1=C(C=C(C2=C1OC(=C(C2=O)O)C3=CC=C(C=C3)O)O)O |
| GJ2 | 6436630 | Alpha-Glyceryl linoleate | CCCCCC=CCC=CCCCCCCCC(=O)OCC(CO)O |
| SG1 | 5281628 | Hispidulin | COC1=C(C2=C(C=C1O)OC(=CC2=O)C3=CC=C(C=C3)O)O |
| HP | 34365085 | l-Isoequilenin | CC12CCC3=C(C1CCC2=O)C=CC4=C3C=CC(=C4)O |
| GZ3 | 712316 | (-)-Taxifolin | C1=CC(=C(C=C1C2C(C(=O)C3=C(C=C(C=C3O2)O)O)O)O)O |
| GZ2 | 439533 | Taxifolin | C1=CC(=C(C=C1C2C(C(=O)C3=C(C=C(C=C3O2)O)O)O)O)O |
| GZ1 | 182232 | (+)-Epicatechin | C1C(C(OC2=CC(=CC(=C21)O)O)C3=CC(=C(C=C3)O)O)O |
| TR1 | 12358798 | Campesterol | CC(C)C(C)CCC(C)C1CCC2C1(CCC3C2CC=C4C3(CCC(C4)O)C)C |
| TLZ2 | 5282805 | 11,14-Eicosadienoic acid | CCCCCC=CCC=CCCCCCCCCCC(=O)O |
| TLZ1 | 5312529 | 11,14,17-Eicosatrienoic acid | CCC=CCC=CCC=CCCCCCCCCCC(=O)O |
| QL | 56605412 | 4-Oxa-1-azabicyclo[4.2.0]octan-5-one,7-(iodomethyl)-2-phenyl-6-(trifluoromethyl)-, (2S)- | C1C(C2(N1C(COC2=O)C3=CC=CC=C3)C(F)(F)F)CI |
| XS1 | 5359596 | Arsenic | As |
| XS2 | 24434 | Potassium nitrate | [N+](=O)([O-])[O-]·[K+] |

BJ: Biejia; EJ: Ejiao; DH: Dahuang; TBC: Tubiechong; HP: Houpo; GJ: Ganjiang; SW: Shiwei; DS: Danshen; BS: Baishao; MDP: Mudanpi; SF: Shufu; LXH: Lingxiaohua; QM: Qumai; TR: Taoren; CH: Chaihu; QL: Qianglang; TLZ: Tinglizi; SG: Shegan; GZ: Guizhi; BX: Banxia; HQ: Huangqin; FF: Fengfang; XS: Xiaoshi. “A1” represents the common active ingredient of Biejia, Ejiao and Tubiechong; “ B1” represents the common active ingredient of Biejia and Ejiao; “ C1” represents the common active ingredient of Shiwei, Lingxiaohua, Banxia, Baishao, Dahuang, Ganjiang, Guizhi, Taoren and Tinglizi; “D1” represents the common active ingredient of Shiwei, Chaihu, Mudanpi and Tinglizi; “E1” represents the common active ingredient of Shiwei, Baishao, Chaihu, Mudanpi and Tinglizi; “F1” represents the common active ingredient of Banxia, Chaihu and Shegan; “G1” represents the common active ingredient of Baishao and Mudanpi; “H1” represents the common active ingredient of Baishao, Ganjiang, Mudanpi and Guizhi; “I1” represents the common active ingredient of Baishao, Mudanpi and Guizhi; “J1” represents the common active ingredient of Chaihu, Shegan and Tinglizi; “K1” represents the common active ingredient of Shegan and Danshen.
